# FTY720 Regulates Mitochondria Biogenesis in Dendritic Cells to Prevent Kidney Ischemic Reperfusion Injury

**DOI:** 10.3389/fimmu.2020.01278

**Published:** 2020-06-23

**Authors:** Thomas V. Rousselle, Canan Kuscu, Cem Kuscu, Kailo Schlegel, LiPing Huang, Maria Namwanje, James D. Eason, Liza Makowski, Daniel Maluf, Valeria Mas, Amandeep Bajwa

**Affiliations:** ^1^Transplant Research Institute, James D. Eason Transplant Institute, Department of Surgery, School of Medicine, University of Tennessee Health Sciences Center, Memphis, TN, United States; ^2^Division of Nephrology and the Center for Immunity, Inflammation and Regenerative Medicine, Department of Medicine, University of Virginia, Charlottesville, VA, United States; ^3^Department of Pediatrics and Genetics, University of Tennessee Health Science Center, Memphis, TN, United States; ^4^Department of Medicine - Division of Hematology and Oncology, College of Medicine, Department of Pharmaceutical Sciences, College of Pharmacy, University of Tennessee Health Sciences Center, Memphis, TN, United States

**Keywords:** dendritic cell, FTY720, mitochondria, sphingosine-1-phosphate receptor, macrophages, metabolism, acute kidney injury, ischemic reperfusion injury

## Abstract

Dendritic cells (DCs) are central in regulating immune responses of kidney ischemia-reperfusion injury (IRI), and strategies to alter DC function may provide new therapeutic opportunities. Sphingosine 1-phosphate (S1P) modulates immunity through binding to its receptors (S1P1-5), and protection from kidney IRI occurs in mice treated with S1PR agonist, FTY720 (FTY). We tested if *ex vivo* propagation of DCs with FTY could be used as cellular therapy to limit the off-target effects associated with systemic FTY administration in kidney IRI. DCs have the ability of regulate innate and adaptive responses and we posited that treatment of DC with FTY may underlie improvements in kidney IRI. Herein, it was observed that treatment of bone marrow derived dendritic cells (BMDCs) with FTY induced mitochondrial biogenesis, FTY-treated BMDCs (FTY-DCs) showed significantly higher oxygen consumption rate and ATP production compared to vehicle treated BMDCs (Veh-DCs). Adoptive transfer of FTY-DCs to mice 24 h before or 4 h after IRI significantly protected the kidneys from injury compared to mice treated with Veh-DCs. Additionally, allogeneic adoptive transfer of C57BL/6J FTY-DCs into BALB/c mice equally protected the kidneys from IRI. FTY-DCs propagated from *S1pr1*-deficient DCs derived from *CD11cCreS1pr1*^*fl*/*fl*^ mice as well as blunting mitochondrial oxidation in wildtype (WT) FTY-DCs prior to transfer abrogated the protection observed by FTY-DCs. We queried if DC mitochondrial content alters kidney responses after IRI, a novel but little studied phenomenon shown to be integral to regulation of the immune response. Transfer of mitochondria rich FTY-DCs protects kidneys from IRI as transferred FTY-DCs donated their mitochondria to recipient splenocytes (i.e., macrophages) and prior splenectomy abrogated this protection. Adoptive transfer of FTY-DCs either prior to or after ischemic injury protects kidneys from IRI demonstrating a potent role for donor DC-mitochondria in FTY's efficacy. This is the first evidence, to our knowledge, that DCs have the potential to protect against kidney injury by donating mitochondria to splenic macrophages to alter their bioenergetics thus making them anti-inflammatory. In conclusion, the results support that *ex vivo* FTY720-induction of the regulatory DC phenotype could have therapeutic relevance that can be preventively infused to reduce acute kidney injury.

## Introduction

The pathogenesis of kidney injury following kidney ischemia reperfusion (IR) involves a complex interaction between altered microcirculatory hemodynamics, renal parenchymal cells (endothelial and epithelial) and infiltrating immune cells (1, 2). Dendritic cells (DCs), the major leucocyte subset in the kidney (3–5), contributes to both the innate and adaptive immunity of kidney IR injury (IRI) (6) through aberrant activation of immune cells (7–9). Considerable data supports that the immune system mediates acute kidney injury (AKI) (10), yet many of the underlying mechanisms still remain unclear. In preclinical mouse models, anti-inflammatory pharmacologic treatments have been shown to significantly attenuate tissue injury and loss of function (11–14). However, the side effects of these common anti-inflammatory therapies combined with the lack of clinical data, supporting the involvement of the immune system in AKI pathogenesis, have hindered the development of clinically tenable anti-inflammatory options. Therefore, as of now dialysis remains the only treatment option available to AKI patients, underscoring the need to develop novel approaches to tackle this hurdle to ultimately improve patient quality of life.

Our previously published work using mouse models of AKI (13, 14) and others (15, 16) have demonstrated that modulation of Sphingosine 1 Phosphate receptors (S1PRs) significantly influences AKI development and thus progression to chronic kidney injury. These receptors belong to a family of five G-protein coupled receptors (S1pr1-5) that modulate diverse physiological responses including “cellular growth and proliferation, angiogenesis, apoptosis, and lymphocyte trafficking” (17–20). Similar levels of S1PRs are expressed on both human and mouse leukocytes (21–23). FTY720, a potent immunosuppressant and a synthetic S1P agonist is currently in clinical trials for treatment of autoimmune diseases (24) and is effective in reducing graft rejection in preclinical mouse models (25, 26) because it mediates a potent immunosuppression. Phosphorylated-FTY720 (FTY720-P), the active form of FTY720, is a non-selective S1P analog that binds and activates four (S1PR1, 3–5) of the five known receptors for S1P (24, 27). FTY720-dependent protection or diminished disease severity has been demonstrated in varied acute and chronic disease models, such as diabetes (28–33), multiple sclerosis [MS, review (34)], ischemic injury (35–46), and even clearance of viral infection (47). To date, FTY720 is currently used as FDA-approved treatment (Gilenya) of MS patients (48). In our previously published work, we have shown that this pan-S1PR agonist, FTY720, attenuated kidney IRI by directly activating S1P1 on proximal tubule (PT) cells, independent of its previously known function through binding to S1P1 on B and T cells to induce canonical lymphopenia (14). FTY720 also reduces cisplatin-induced AKI (49). Deletion of S1P1 renderers cultured and kidney PT epithelial cells more susceptible to cisplatin-induced injury (49), whereas overexpression of S1P1 protected PT cells from injury and resistance to cisplatin induced cell death at lower doses (49). One potential mechanism that we previously reported to mediate S1P1 protection in IRI and cisplatin-induced AKI was through possible induction of mitochondrial biogenesis that resulted in higher mitochondria numbers and ultimately preserved kidney function (49). Thus, we previously concluded in these published studies that S1P1 had a central role in stabilizing mitochondrial function and FTY720 administration could represent a novel strategy in the prevention of AKI (14, 49).

However, use of pharmacological agents such as FTY720 has limitations due to off-target (binding to other S1P receptors) and other associated adverse side effects. On the other hand, cell-based therapeutic approaches have advantages; transferred cells are capable of sensing diverse signals, navigating to specific sites in the body, make immunological decisions and executing complex responses. Dendritic cells (DCs) are heterogeneous, professional antigen-presenting cells (APCs) and are distributed throughout the lymphoid and non-lymphoid tissues (50). Our previous studies demonstrated that S1P3 deficient (*S1pr3*^−/−^) mice are protected from renal IRI through a mechanism that involved BMDCs and their ability to respond as immune modulators to regulate innate and adaptive immune responses (13). Additionally, we had also tested the therapeutic advantage of using *S1pr3*^−/−^ BMDC in DCs transfer studies in mouse kidney IRI model. Compared to mice treated with wild-type (WT) DCs that had significant rise in plasma creatinine, mice that received *S1pr3*^−/−^ DCs were significantly protected from kidney IRI (12, 13). *S1pr3*^−/−^ DCs did not attenuate IRI in splenectomized, *Rag1*^−/−^, or DC-depleted (*CD11c-DTR*) mice (12) demonstrating that both spleen derived cells, likely macrophages (CD169^+^ or F4/80^+^) or DCs (CD11c^+^ or CD103^+^) and T cells (CD4^+^ and Tregs) mediated this protection.

The aim of this study was to determine the potential protective mechanism(s) of FTY720 stimulated BMDCs in a preclinical mouse model of kidney IRI. Treatment of BMDCs *ex vivo* with FTY720 avoids any adverse off-target effects associated with systemic drug injections. Herein, we demonstrate that FTY720 treated BMDCs (FTY-DC) accumulate in the recipient spleen as early as 30 min after adoptive transfer of cells via intravenous injection. FTY-DC mitochondrial content was elevated *in vitro*, and we posit that transfer of FTY-DC mitochondria to splenic macrophages occurs. Indeed, in spleen, FTY-DC interaction with splenic macrophages (CD169^+^ and F4/80^+^) was evident. Transplant of mitochondria from FTY-DC reprogrammed macrophage phenotype; macrophages were less immunogenic upon inflammatory stimuli *in vivo* and *in vitro*. Depletion of DC-derived mitochondria through varied approached demonstrated that oxidative capacity of DC was critical to protection from AKI in response to IRI. Splenectomy or pharmacologic ablation of mitochondrial function with combination treatment with rotenone and antimycin A (Rot/AA) of FTY-DC abrogated the protection observed with FTY-DCs. Likewise, inhibiting FYT720 agonism using S1P1 receptor deficient DCs (*CD11cCreS1pr1*^*fl*/*fl*^) also reversed FTY-DC therapeutic efficacy. Overall, the interactions between FTY-DC and splenocytes (macrophage) demonstrated that induction of the anti-inflammatory or immunosuppressive phenotype led to reduced injury, an effect that required the recipient spleen. Of note, adoptive transfer of DC worked equally well in allogeneic IRI model (C57BL/6 BMDC → BALB/c mice), suggesting that this cell-based therapy can be efficacious in transplantation. Finally, we provide seminal findings that DCs are mitochondrial donors which illustrate a novel mechanism of how DCs regulate innate immune responses in acute injury.

## Materials and Methods

### Mice

All animals were handled, and procedures were performed in adherence to the National Institutes of Health Guide for the Care and Use of Laboratory Animals, and all protocols were approved by the University of Tennessee Health Science Center and University of Virginia Institutional Animal Care and Use Committees. *CD11cCre* mice (Jackson Laboratories, Bar Harbor, ME) were purchased and *S1pr1*^*fl*/*fl*^ generously provided by Dr. Richard L. Proia, NIH. The lines were crossed and bred as fl/fl with Cre to generate *CD11c*Cre*S1pr1*^*wt*/*wt*^ (control) or *CD11c*Cre*S1pr*^*fl*/*fl*^ (DC specific S1pr1 knockout) littermates. *Pham*^fl/fl^ mice (51) (Jackson Laboratories, Bar Harbor, ME) were bred with CD11cCre to obtain *CD11c*Cre*Pham*^*fl*/*fl*^ mice. For all transfer studies C57BL/6J and BALB/c mice were purchased from the National Cancer Institute, NCI (Frederick, MD). Mice were maintained in standard vivarium housing with a 12 h light/dark cycle on a chow diet and water was freely available.

### Renal Ischemia-Reperfusion Injury and Splenectomy (SPLNX)

Mice were anesthetized with an intraperitoneal injection of a ketamine (120 mg/kg) and xylazine (12 mg/kg) mixture and buprenorphine (0.15 mg/kg, subcutaneous injection) was administered as an analgesic and placed on a warm pad to maintain body temperature at 34.5–36°C. Mice were then randomized to sham or IRI operation. Bilateral flank incision was performed and either the renal vessels (vein and artery) on both sides or only on the left side were cross-clamped. Body temperature was checked and maintained throughout the ischemic period using ATC-2000 system (World Precision Instruments, Sarasota, FL). Sham-operated mice underwent the same procedure except for vessel clamping and surgical wounds were closed. Male mice (8–12 wk old, C57BL/6 and BALB/c) were subjected to bilateral IRI (26 min ischemia for C57BL/6 and 28 min for BALB/c mice followed by 20–24 h reperfusion) as previously described (3, 7, 52). Mice that had one kidney with no reperfusion 24 h after ischemia were excluded from all analysis. For experiments that involved splenectomy (Splnx) prior to IRI, mice were anesthetized with an intraperitoneal injection of ketamine (120 mg/kg) and xylazine (12 mg/kg). The spleen was then removed through a small flank incision. Control, sham-operated mice underwent the same procedure except for splenic artery ligation and spleen removal. Sham and splenectomized mice recovered for 7 days prior to BMDC transfer for IRI studies.

### Assessment of Kidney Function and Histology

Blood was collected under anesthesia from the retro-orbital sinus, and plasma creatinine (mg/dL) was determined by using an enzymatic method with minor modifications from the manufacturer's protocol (twice the volume of sample; Diazyme Laboratories, Poway, CA) and as previously reported (53). For histology, kidneys were fixed overnight in 0.2% sodium periodate-1.4% DL-lysine-4% paraformaldehyde in 0.1 M phosphate buffer, pH 7.4 (4% PLP) and embedded in paraffin. Kidneys were prepared for Hematoxylin and eosin (H&E) staining as previously described (3) and viewed by light microscopy (Zeiss AxioSkop). Photographs were taken and brightness/contrast adjustment was made with a SPOT RT camera (software version 3.3; Diagnostic Instruments, Sterling Heights, MI). For quantification of tubular injury score, sections were assessed by counting the percentage of tubules that displayed cell necrosis, loss of brush border, cast formation, and tubule dilation as follows: 0 = normal; 1 = <10%; 2 = 10 to 25%; 3 = 26 to 50%; 4 = 51 to 75%; 5 = >75%. Five to 10 fields from each outer medulla were evaluated and scored in a blinded manner. The histological change was expressed as acute tubular necrosis (ATN), scored as previously described (13, 54).

### Immunohistochemical Analysis

Kidneys were fixed in 1% PLP (as above except 1% paraformaldehyde) overnight, incubated in 30% sucrose for 24 h at 4° C, and embedded and frozen in Tissue-Tek OCT Compound (Ted Pella Inc., Redding, CA). Frozen sections (5–7 μm) were permeabilized with 0.3% Triton X-100, and non-specific binding was blocked with 10% horse serum and rat anti-mouse CD16/32 (10 μg/ml; clone 2.4G2; BD Pharmingen, San Jose, CA). Sections were labeled by incubation for 1 h with anti-mouse F4/80 (5 μg/ml; clone BM8, Molecular probes, Fredrick, MD), anti-mouse CD169 (7 μg/ml; clone 3D6.112, BioLegend, San Diego, CA), anti-mouse CD169 (7 μg/ml; clone MOMA-1; AbD Serotec/BioRad, Raleigh, NC). All specimens were mounted with ProLong Gold Antifade reagent with DAPI (Invitrogen, Carlsbad, CA) to label cell nuclei. Images were acquired using a Zeiss Axiovert 200 microscopy system with ApoTome imaging and Axiovision 4.8 software (Carl Zeiss Microscopy, Thornwood, NY).

### Bone Marrow (BM)-Derived-Dendritic Cell (DC) Culture and Adoptive Transfer

Eight week old C57BL/6J WT, *CD11c*Cre*S1pr*^fl/fl^, *CD11c*Cre*Pham*^fl/fl^ *S1pr3*^−/−^ male mice were used for generating DCs from whole BM precursors (55). GMCSF-rich supernatant was derived from J558L cells stably transfected with mouse GMCSF. The cell line was a generous gift from Dr. Ira Mellman (Dept. of Biology, Yale University). Briefly, freshly isolated BM was cultured with 6 ng/ml recombinant mouse GMCSF (total of 3 treatments) for 8 days in RPMI 1640 (Invitrogen). Eighty to Ninety percentage of resulting cells were CD11c^+^ DCs as determined by flow cytometry with CD11c antibody. The optimal dose of 1 μM FTY720 was decided after testing various doses (0.1–10 μM). Similar to studies done by Zeng et al. (56) FTY720 treated BMDCs were tested for drug induced cellular toxicity (apoptosis) and changes in co-stimulatory molecules (CD40, CD80, CD86, and MHCII) after overnight treatment with LPS. BMDC were treated with 1 μM FTY720 (total of 4 treatments) that was purchased from Cayman Chemicals (Ann Arbor, Michigan). BM-derived DCs were treated with TLR4 agonist lipopolysaccharide (LPS; *Escherichia coli* serotype 0111:B4; 25 or 100 ng/mL; Sigma-Aldrich); or vehicle (1x PBS) for 24 h in culture medium for syngeneic studies (C57BL/6J BMDCs→ C57BL/6J mice) and left untreated for allogenic studies (C57BL/6J BMDCs→ BALB/c mice). See timeline in Supplemental Figure 1. Cells were washed, and 0.5 × 10^6^ cells per mouse were i.v. injected to naive mice 1 day before or in some studies 4 h after kidney IRI. Griess Reagent system (Promega) was used to detect nitrate in media after LPS stimulation. BMDCs were labeled with MitoTracker CMXRos Red or MitoTracker Green (50–100 nM, 30 min @ 37°C, Invitrogen) or Mitosox (5 μM; 10 min @ 37°C; Invitrogen) prior to fixing with 4% paraformaldehyde in 0.1 M phosphate buffer, pH 7.4 for 30 min. Actin was labeled with phalloidin-FITC (2.5 μg/ml; Sigma). Nuclei were visualized using DAPI. All specimens were mounted with ProLong Gold Antifade reagent with DAPI (Invitrogen). Images were acquired using the Zeiss Axiovert 200 microscopy system with ApoTome imaging and Axiovision software (Carl Zeiss Microscopy LLC, Thornwood, NY). RAW264.7 cells were purchased from ATCC (Manassas, VA) and maintained in DMEM (Invitrogen).

### Quantitative Real-Time PCR

Total RNA was isolated and reversed transcribed to cDNA, and RT-PCR was performed as previously described (13, 49, 57). Primers span an exon-exon junction and were designed with Primer-BLAST (NCBI). *Pgc1a*, NM_008904.2, 5′GCTCTTCCTTTAACTCTCCGTGTC3′ and 5′CTTGACCTGGAATAT GGTGATCGG (53). Relative mitochondrial DNA (mtDNA) expression level was measured as previously described (58). Briefly, total genomic DNA was isolated and equal amounts (5 ng) was used for RTPCR using ND1 as surrogate primers for mtDNA and HK2 primers for nuclear DNA (nDNA) for mouse and ND6 for human mtDNA as previously described (58, 59). Total number of mtDNA copies was determined by following formula, delta Ct = nDNA gene (Ct)- mtDNA (Ct); mtDNA copy = 2 × 2^δ^*Ct* (58).

### Mitochondria Isolation and Quantification

Mitochondria were isolated from mouse liver or BMDCs as previously described (60). Briefly, 2 pieces of ~6 mm mouse liver biopsies were homogenized using homogenization buffer (300 mmol/L sucrose, 10 mmol/L HEPES-KOH, 1 mmol/L EGTA-KOH, pH 7.4) in a C tube (Miltenyi Biotec, Cambridge, MA) with GentlyMACS dissociator using the “m-mito tissue” pre-set program. The homogenate was incubated on ice for 10 min with 1 mg Subtilisin A protease from *Bacillus licheniformis* (Sigma-Aldrich, St. Louis, MO). The digested homogenate was serially filtered through 2 × 40 μm Falcon Cell Strainers (Thermo-Fisher, Waltham, MA) and 1 × 10 μm PluriSelect mesh (PluriSelect, San Diego, CA) that was saturated with ice cold homogenization buffer. Mitochondria were collected by centrifuging the filtrate at 3,500 × g at 4°C for 10 min and re-suspended in cold 1x PBS for further use. Protein concentration of isolated mitochondria were determined using Bradford assay according to manufacture recommendations. Isolated mitochondria were kept on ice and used within 1 h after isolation. In some experiments isolated mitochondria were sonicated and kept on ice before injecting, all isolated mitochondria were injected within 1 h of isolation. ATP concentrations of isolated mitochondria were using luminescent CellTiter-Glo reagent (Promega) according to the manufacturer's instructions. Isolated mitochondria were injected (i.v.; 0–100 μg/mouse) 1 day before spleen was harvested for single cells preparation for *in vitro* stimulation with LPS (100 ng/ml) for 6 h. RAW264.7 cells (TIB-71, ATCC, Old Town Manassas, VA) were treated with isolated mitochondria (with and without sonication, 10 μg/ml) from DCs for 24 h before stimulating with LPS (100 ng/ml) or analysis with Seahorse Bioanalyzer (Agilent Technologies, Santa Clara, CA).

### Seahorse Flux Bioanalyzer

Seven day old BMDCs were transferred to a Seahorse 24-well tissue culture plates and oxygen consumption rate (OCR) was measured, and parameters were calculated as previously described (49) with the following modification. Prior to the assay, the media was changed to unbuffered DMEM (Gibco #12800-017, pH 7.4, 37°C), and cells were equilibrated for 30 min at 37°C. After measuring basal respiratory rate, Oligomycin (Sigma; 2 μM; uncouples ATP-coupled respiration by inhibiting ATP synthase), FCCP (Sigma; 1.5 μM; carbonyl cyanide 4-(trifluoromethoxy)-phenylhydrazone (FCCP), mitochondrial uncoupling agent; uncouples mitochondrial respiration from ATP to determine maximal respiratory rate), and electron transport chain (complex I and III) inhibitors, rotenone (Sigma; 0.5 μM) and antimycin A (Sigma; 0.5 μM; to eliminate all mitochondrial respiration) were injected sequentially during the assay. OCR was measured in 3 min periods of time (over a total period of 2 h). Basal mitochondrial respiration, ATP-linked respiration, proton leak (non-ATP linked oxygen consumption), maximal respiration, non-mitochondrial respiration, reserve respiratory capacity, respiratory control ratio, and coupling efficiency were determined in whole cells according to Brand et al. (61), *N* = 4–5 wells were used for each experimental group and experiments were repeated a minimum of 3 times.

### Flow Cytometric Analysis, Western Blot, ELISA, and 32-Plex Luminex

Flow cytometry was used to analyze kidney leukocyte content. In brief, kidneys were extracted, minced, and digested (1 mg/ml collagenase) as described (7). After blocking nonspecific Fc binding with anti-mouse CD16/32 (2.4G2), fresh kidney suspensions were incubated with fluorophore-tagged anti-mouse CD45 (30-F11) to determine total leukocyte cell numbers. CD45-labeled samples were further used for labeling with different combinations of fluorophore-tagged anti-mouse F4/80 (BM8), GR-1 (Ly6G), CD11b (M1/70), CD11c (integrin alpha X chain-HL3). 7-AAD (BD Biosciences) was added 15 min before analyzing the sample to separate live from dead cells. Appropriate fluorochrome-conjugated, isotype-matched, irrelevant mAbs were used as negative controls. Flow cytometry data acquisition was performed on a FACS Calibur (Becton Dickinson, San Jose, CA) with Cytek 8-color flow cytometry upgrade (Cytek Development, Inc., Fremont, CA). Data were analyzed by FlowJo software 9.0 (Tree Star, Ashland, OR). All antibodies (except as noted) were from eBioscience and were used at a concentration of 5 μg/ml. ELISA: Media was collected from BMDCs or splenocytes treated for 6 or 24 h with wither 25 or 100 ng/ml LPS. TNFα levels were measured by using mouse ELISA kits (Invitrogen, Carlsbad, CA) following the manufacturer's protocol. BMDCs treated with and without LPS for 24 h were used to isolated total protein using RIPA lysis buffer supplemented with protease and phosphatase inhibitor cocktail (Thermo Fisher Scientific, Vernon Hills, IL). Equal volumes of the lysate supernatants were either boiled for 10 min at 100°C for GAPDH) or left at room temperature for 10 min for rodent OXPHOS cocktail (Abcam, Cambridge, MA) with Laemmli buffer and β-mercaptoethanol. Total of 20 μg of proteins were separated using a 10% SDS-PAGE gel and transferred to PVDF membranes. PVDF membranes were incubated overnight with primary antibody for GAPDH (1:1000, Santa Cruz Biotechnology) and rodent OXPHOS cocktail (1:1000). Blots were then washed and incubated at 1:4000 for 1 h with horseradish peroxidase-conjugated anti-mouse secondary antibodies (Santa Cruz Biotechnology). Bands were visualized by chemiluminescence according to the manufacturer's protocol with SuperSignal West Pico chemiluminescent substrate (Thermo Fisher Scientific) and quantified by Image J. The Bio-Plex Pro Mouse Cytokine 23-Plex Immunoassay was used to check serum levels 24 h after bilateral kidney IRI (BioRad, Hercules CA).

### Data and Statistical Analysis

GraphPad Prism 8 (GraphPad Inc.), SigmaPlot 11.0 (Systat Software Inc.), and Canvas X (ACD Systems of America Inc.) were used to analyze and present the data. Data were analyzed, after transformation if needed to generate a normal distribution, by 2-tailed *t*-test or 1-way ANOVA with *post-hoc* analysis as appropriate. Two-tailed unpaired *t*-test was used for analysis of two groups. *p* < 0.05 was used to indicate significance.

## Results

### FTY720 Induces Metabolic Reprogramming in WT BMDCs

**Figure 1:**
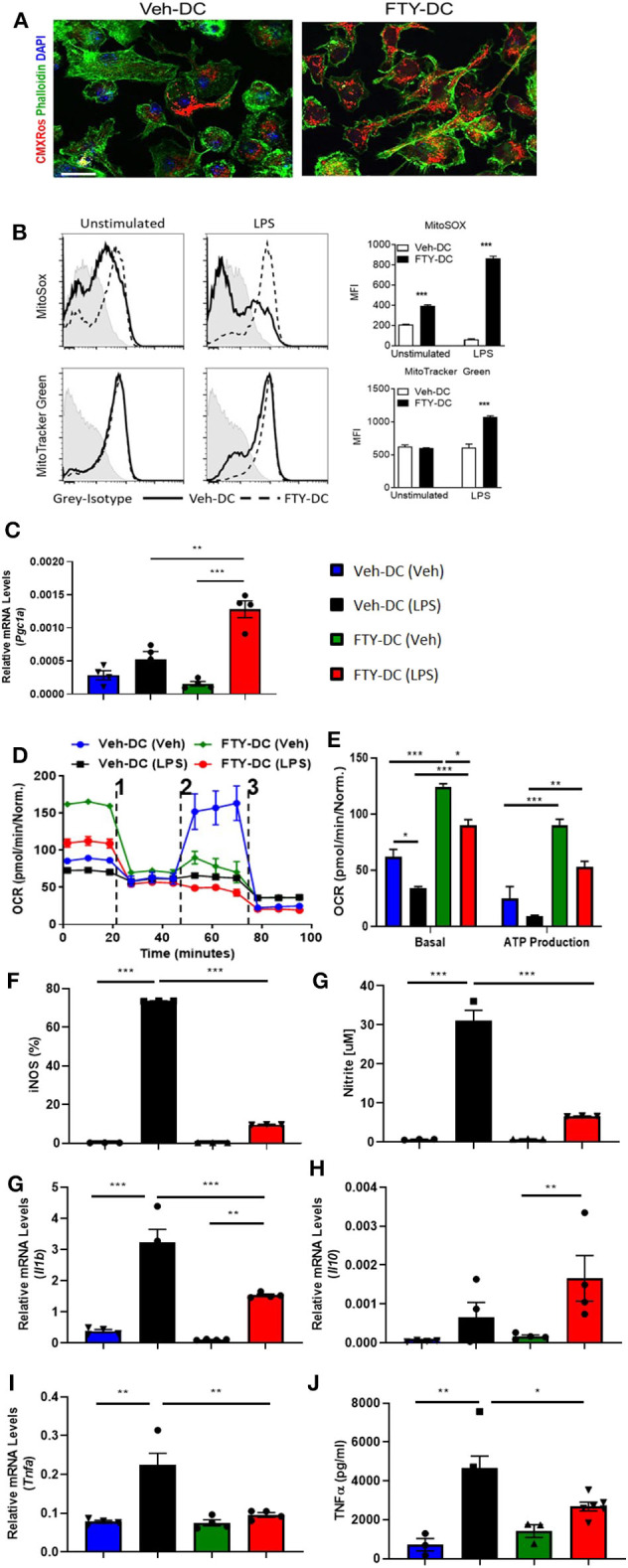
FTY720 treatment induces less immunogenic DCs that have higher mitochondria numbers. **(A)** 8 day old Veh-DC and FTY-DC were labeled with MitoTracker CMXRos (50 nM). Scale bar, 20 μm. **(B)** Veh-DC and FTY-DC treated with 100 ng/ml LPS for 24 h or left unstimulated (unstim). Levels of MitoSox (5 μM) and MitoTracker green (100 nM) were measured after 24 h of LPS treatment. **(D–E)** DCs were seeded in a Seahorse XF-24e analyzer, stimulated with and without LPS for 24 h, and oxygen consumption rate (OCR) was determined during sequential treatments with Oligomycin (1), FCCP (2) and antimycin A plus rotenone (3). Quantification of basal OCR and ATP production. **(F)** Flow cytometry analysis of iNOS expression was determined with and without LPS stimulation. Quantification of percent of iNOS in Veh-DC and FTY-DC after LPS. **(G)** Nitrite levels were determined in culture supernatants with and without LPS stimulation. mRNA levels of *Pgc1a* **(C)***, Il1b* **(H)***, Il10* **(I)**, and *Tnfa* **(J)** in Veh-DC and FTY-DC treated with 100 ng/ml LPS or left unstimulated for 24 h. **(K)** ELISA of TNFα from the Veh-DC and FTY-DC treated with 100 ng/ml LPS for 24 h. *p ≤ 0.05, **p ≤ 0.01, and ***p ≤ 0.001, one-way ANOVA followed by Tukey's post-test. Data represent means ± SEM of triplicates. One of three experiments is shown.

WT DCs were isolated and propagated for 8 days in presence of GMCSF and vehicle (1X PBS) or FTY720 (1 μM). Eight day old DCs were labeled with MitoTracker CMXRos Red (50 nM). Compared to vehicle treatment, FTY720 treatment increased mitochondrial content in BMDCs (Figure 1A). Similarly, there was significantly higher labeling for MitoSox (5 μM) and MitoTracker Green (100 nM) after over-night LPS stimulation in FTY-DC compared to Veh-DC (Figure 1B). FTY-DCs displayed significantly elevated mRNA levels for peroxisome proliferator-activated receptor gamma co-activator 1-alpha (*Pgc1a*) in response to LPS, but this LPS-induction was absent in Veh controls (Figure 1C). To determine if the changes in mitochondrial content also altered mitochondrial function, bioenergetic analysis was undertaken. LPS blunted oxygen consumption compared to Veh controls in Veh-DCs as expected (Figure 1D, blue to black line) (62). Interestingly, FTY-DCs have higher basal OCR (Figure 1D, green to blue line at time zero). Upon treatment with uncoupler FCCP, FTY-DC demonstrate a failure to increase maximal respiratory capacity in unstimulated cells that is even more reduced with LPS stimulation demonstrating that FTY ablates spare respiratory capacity (likely because already at maximal OCR in basal state). When ATP production was quantified, LPS reduced ATP production as measured by OCR (blue to black). FTY-DC demonstrated significantly greater ATP production compared to Veh-DC in both unstimulated and LPS-stimulated DCs (Figure 1E). These data indicate that propagation of BMDCs in presence of FTY720 increased mitochondrial content, basal OCR, and ATP production. This suggests the potential for an anti-inflammatory phenotype in DCs.

### FTY720 Induces Immune Reprogramming in WT BMDCs

LPS stimulation of BMDCs increased expression of enzymes (iNOS) and cytokines typical of pro-inflammatory DCs. FTY-DC dramatically blunted LPS-induced iNOS expression and nitrate in media compared to Veh-DC (Figures 1F,G). Likewise, FTY significantly blunted expression of LPS-induced *Il1b* and *Tnfa* and protein concentrations of TNFα compared Veh-DC treated with LPS (Figures 1H–K). *Il12p40* gene expression was not regulated by FTY (data not shown). In contrast, *Il10*, a cytokine often associated with anti-inflammatory immune cells was significantly increased by LPS but only in FTY treated cells (Figure 1I). Interestingly, after LPS treatment, FTY-DC have significantly lower expression levels of co-stimulatory antigen presentation molecules (CD80, CD86, and CD40) and MHCII compared to Veh-DC (Supplemental Figure 1). Interestingly, FTY-DCs had lower expression level for PDL1 compared to Veh-DCs and maintained the PDL1/CD86 ratio after LPS stimulation compared to LPS treated Veh-DCs (Supplemental Figure 1). No significant changes in CD11c expression was observed between Veh- and FTY-DCs, although FTY-DC had lower side scatter signal indicating smaller size of cells after LPS stimulation (data not shown). FTY-DCs had higher relative mtDNA levels compared to Veh-DCs (106 ± 13.03 vs. 165.9 ± 20.6). Additionally, we measured total protein changes in mitochondrial OXPHOS complexes (I-V) in Veh- and FTY-DCs treated overnight with 100 ng/ml LPS. FTY-DCs have higher protein levels of different mitochondrial complexes compared to Veh-DCs with and without LPS treatment (Supplemental Figures 1H–J) especially in levels of complex IV (MTCO1) a protein that is encoded by mitochondrial DNA (mtDNA). Mitochondria complex IV was significantly higher in vehicle treated FTY-DC compared to vehicle treated Veh-DC (0.10 ± 0.01 vs. 0.49 ± 0.01, *p* < 0.01) and after LPS treatment (0.04 ± 0.003 vs. 0.16 ± 0.01, *p* < 0.01) along with higher complex III after LPS treatment (Supplemental Figure 1J).

### Transfer of FTY-DC Protects Kidneys From Ischemic Injury

**Figure 2:**
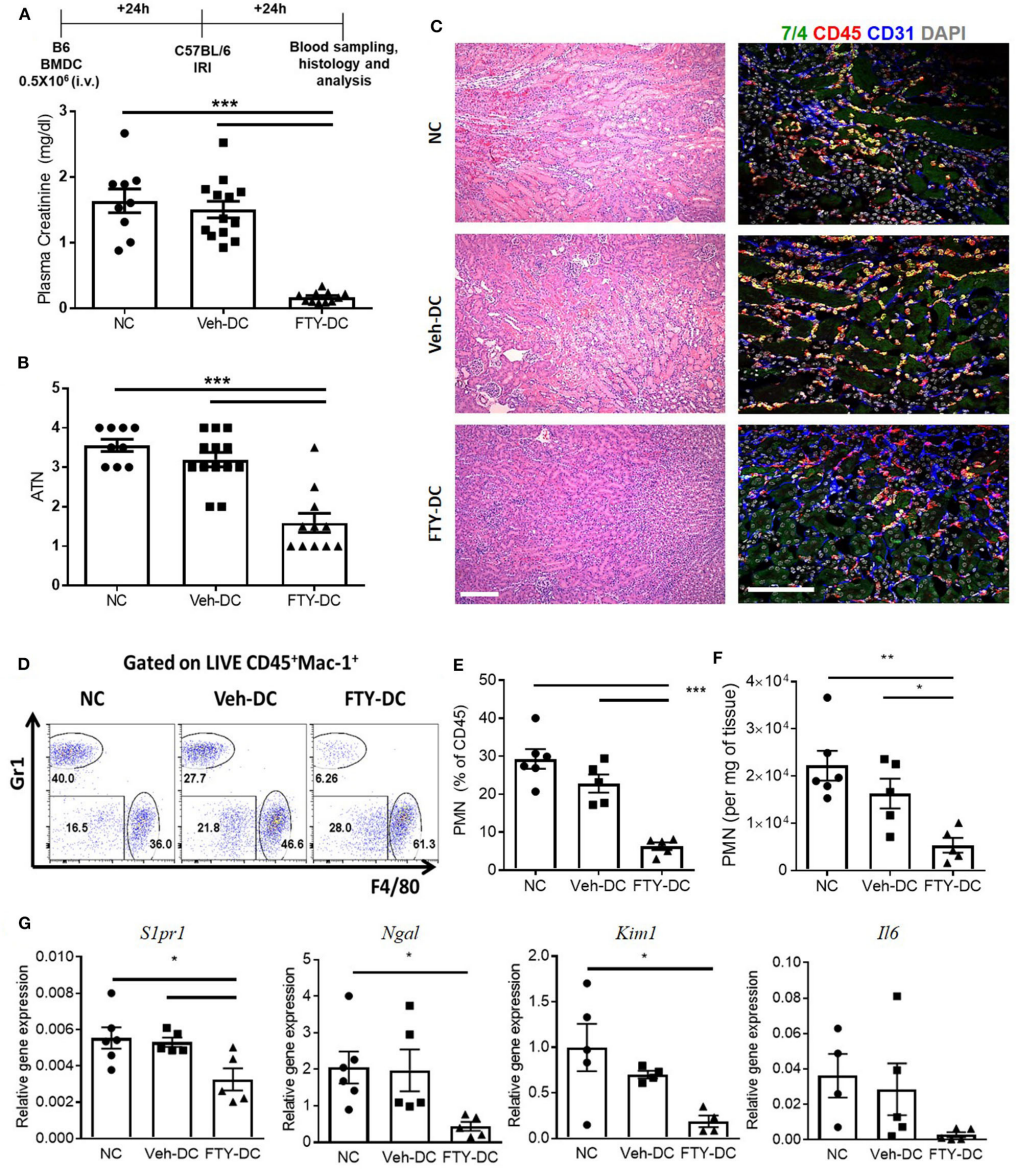
Pretreatment with FTY-DCs protects kidneys from ischemia reperfusion injury. Mice were i.v. injected with 0.5 × 10^6^ DCs (Veh-DC or FTY-DC) and as control no cells (NC) 1 day before bilateral kidney IRI. **(A)** Protocol for experimental setup and plasma creatinine. **(B,C)** Quantification of acute tubular injury (ATN). Renal histology (H&E). Scale bar, 100 μm. **(D–F)** Flow cytometry of kidney tissue gated on neutrophils (CD45^+^Mac-1^+^ Gr1^+^; PMNs) from mice either treated with NC, Veh-DC or FTY-DC. **(G)** Gene expression of kidney *S1pr1, Ngal, Kim1*, and *Il6*. Data represent means ± SEM, *p ≤ 0.05, **p ≤ 0.01, and ***p ≤ 0.001 one-way ANOVA followed by Tukey's post-test.

All DCs were activated with 100 ng/ml LPS prior to transfer in all syngeneic studies (B6 BMDC to B6 mice). Half a million DCs were injected 1 day before bilateral kidney IRI. As control, mice were injected with 1x PBS as no cell (NC) controls. Compared to NC and Veh-DC treated mice, FTY-DC treated mice significantly protected the kidneys from injury (Figure 2A). Morphological changes (Figure 2B) paralleled functional studies. FTY-DC treatment resulted in less infiltration of immune cells (CD45 labeled) compared to Veh-DC or NC treated mice (Figure 2C). Quantitative analysis with flow cytometry further demonstrates that FTY-DC treated mice have few neutrophil infiltrations compared to NC or Veh-DC treated mice (Figures 2D–F). To determine if kidney injury genes along with *S1pr1* were regulated we measured by qRT-PCR relative kidney levels in DC treated mice. Mice treated with FTY-DC have significantly lower kidney mRNA levels for *S1pr1, Ngal, Kim1*, and lower levels for *Il6* (Figure 2G). These data indicate that FTY-DC treated mice have significantly less inflammation (cytokine levels) that results in less infiltration of innate immune cells (PMNs) after kidney IRI. The expression levels of S1pr1 increase after IRI compared to sham operated mice in a time dependent manner (54), possibility indicating initiation of compensatory mechanism due ischemic injury. Plasma samples from Veh- and FTY-DC treated mice were check 24 h after bilateral ischemia using 23-plex Luminex. Only 8 out of 23 cytokines showed low levels of signal. There were no significant changes in circulating levels of MCP-1, IL-9, LIX, or Eotaxin, although trends were toward lower levels in FTY-DC treated mice. Only 3 cytokines were significantly lower (pg/ml; KC [382.06 ± 99.7 vs. 87.22 ± 19.4, *p* < 0.05], IL-1a [508.2 ± 103.1 vs. 245.5 ± 103.4, *p* < 0.05] and G-CSF [910 ± 15.1 vs. 542 ± 135.1, *p* < 0.05]) and significantly higher levels of circulating GM-CSF [46.3 ± 3.6 vs. 112.1 ± 9.8, *p* < 0.05] in FTY-DC treated mice compared to Veh-DC treated mice.

### Injected DCs Transfer Mitochondria to Splenic Macrophages

**Figure 3:**
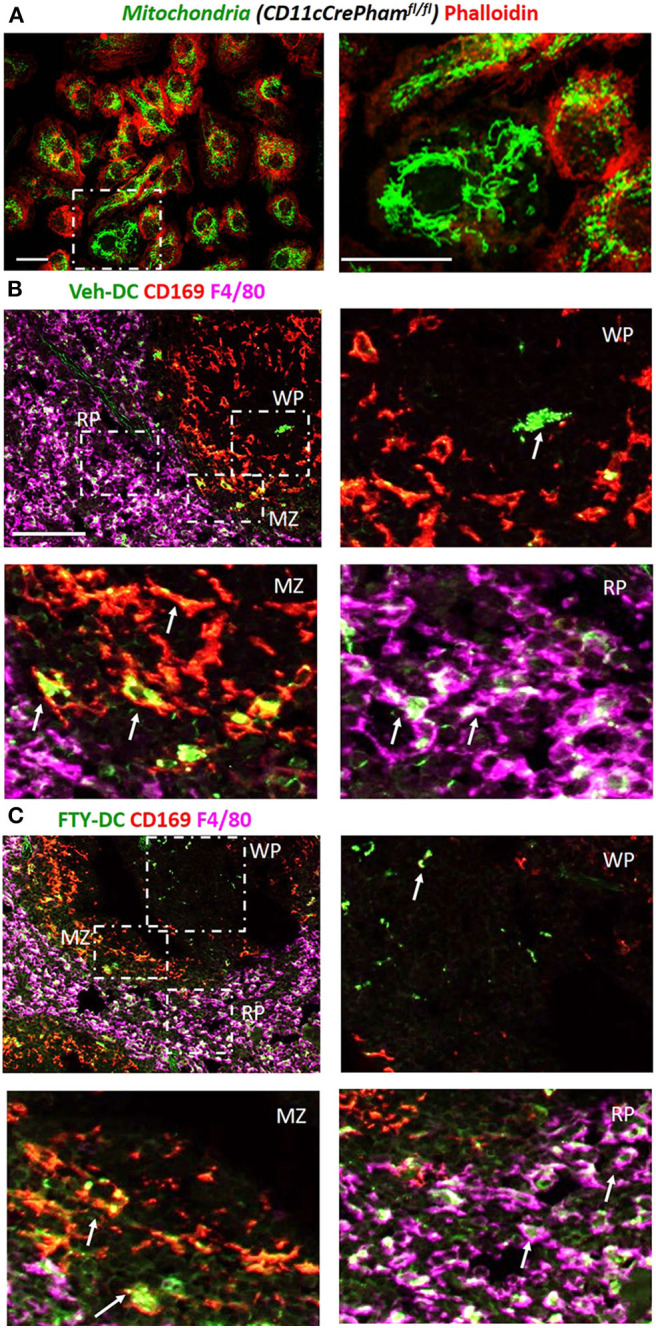
Injected DCs are detected in recipient spleen after adoptive transfer and donate more mitochondria to splenic macrophages. **(A)** BMDCs were propagated from *CD11cCrePham*^*fl*/*fl*^ mice. 8 day old BMDCs were grown on coverslip and labeled with phalloidin (red, actin) and endogenous mitochondria (green), Scale bar, 20 μm; inset on right, Scale bar, 20 μm. **(B)** *CD11cCrePham*^*fl*/*fl*^ Veh-DC and **(C)** FTY-DC labeled with CD169 (red, clone MOMA-1, marginal zone macrophages) and F4/80 (magenta, red pulp macrophages). The appearance of yellow is colocalization of injected mitochondria (green) with CD169 (red) and white is colocalization of injected mitochondria (green) with F4/80 (magenta). RP, red pulp; WP, white pulp; and MZ, marginal zone. Scale bar, 100 μm (top **B,C**). Scale bar, 20 μm (bottom **B,C**). White arrows point to the taken-up mitochondria from *CD11cPham*^*fl*/*fl*^ BMDC.

Next to evaluate if DCs transfer mitochondria to recipient cells we harvested BMDCs from *CD11cCrePham*^*fl*/*fl*^ mice that contain a fluorescent tag in their mitochondria (Figure 3A). Half a million Veh-DC or FTY-DC that were propagated from *CD11cCrePham*^*fl*/*fl*^ mice were i.v. injected and signal in spleen was evaluated 30 min or 24 h after injection. The spleen was labeled with anti-CD169 to identify marginal zone (MZ) and anti-F4/80 for red pulp (RP) macrophages and no antibody labeled area are labeled as white pulp (WP) (Figures 3B,C). Some green fluorescence signal indicative of mitochondria exchange from DCs to macrophages in CD169^+^ cells at the 30 min after injection time point was evident (data not shown). Strong signal in proximity and inside the various splenic macrophages at 24 h from injected DCs is demonstrated (Figures 3B,C). In addition to possibly more mitochondria transfers from FTY-DC, it appears there is disruption in the MZ macrophages (CD169) with FTY-DC treatment along with more mitochondria signal in red pulp compared to Veh-DC (Figure 3C). This disruption in MZ macrophages in mice treated with FTY-DC could possibly be due to similar mechanism that we have previously demonstrated using *S1pr3*^−/−^ DCs; that ultimately result in higher CD4^+^FoxP3^+^ Tregs in white pulp (12).

### Splenectomy (Splnx) Abrogates FTY-DC Dependent Protection

**Figure 4:**
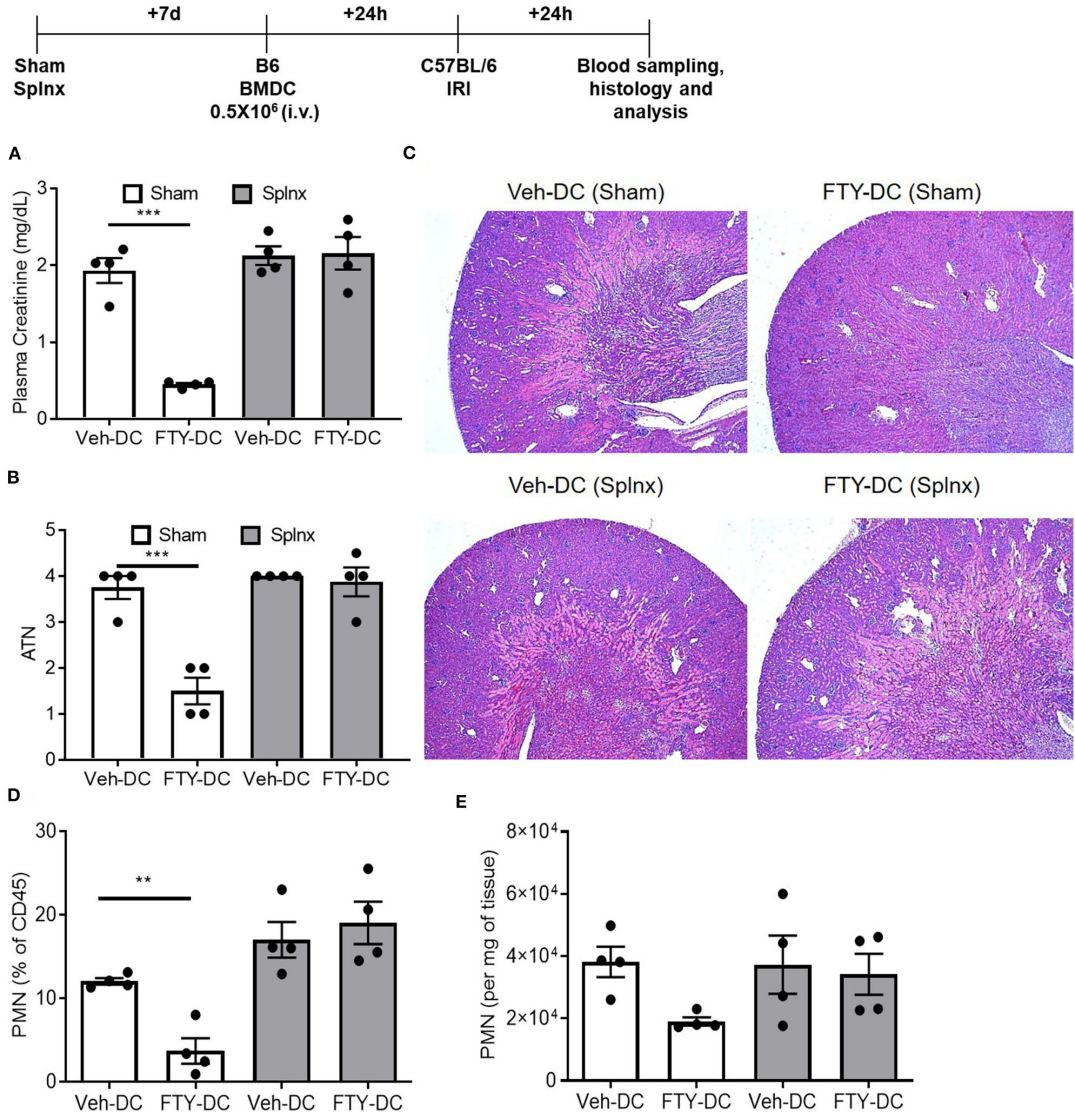
FTY-DC require recipient spleen to protects kidneys from IRI. Protocol for experimental setup. **(A)** Plasma Creatinine (PCr). Mice were i.v. injected with 0.5 × 10^6^ DCs (Veh-DC or FTY-DC) 7 days after splenectomy (Splnx) and 1 day before bilateral kidney IRI. **(B,C)** Quantification of tubular injury. Renal histology (H&E). **(D,E)** Flow cytometry of kidney tissue gated on neutrophils from Sham (no Splnx) mice either treated with Veh-DC or FTY-DC. Data represent means ± SEM, ***p ≤ 0.001, **p ≤ 0.01; one-way ANOVA followed by Tukey's post-test.

Since abundant signal from injected DCs (*CD11cCrePham*^*fl*/*fl*^) was found in spleen, to determine if spleen was important in FTY-DC-dependent protection after kidney IRI, mice underwent either sham or Splnx surgeries and were allowed to recover for 7 days. On day 8, half a million LPS treated either Veh-DC or FTY-DCs were injected 1 day before bilateral kidney IRI, as above. In absence of spleen, FTY-DC dependent protection was completely abrogated (Figure 4A). Histological evaluation also showed dramatic FTY-DC-dependent protection of kidney architecture (Figures 4B,C). Quantitative analysis with flow cytometry further demonstrates that FTY-DC treated Sham mice have few neutrophil infiltrations compared to Veh-DC treated mice (Figures 4D,E), no changes in neutrophil percentage or numbers was observed in Splnx-FTY-DC treated mice. In addition to involvement of innate immune cells (macrophages) as possible mitochondria recipients from injected DCs, it is plausible that DCs could donate mitochondria to other adaptive immune cells (T and/or B cells). Therefore, we evaluated spleens of sham (no Splnx) mice after kidney IRI that were treated with either Veh-DCs or FTY-DCs. FTY-DC treated mice had higher total number of splenic Tregs (CD4^+^Foxp3^+^) compared to Veh-DC treated mice (44,593 ± 7,136 vs. 71,173 ± 10,436, *p* = 0.09).

### Dendritic Cell S1P1 Are Required for FTY-DC Dependent Protection

**Figure 5:**
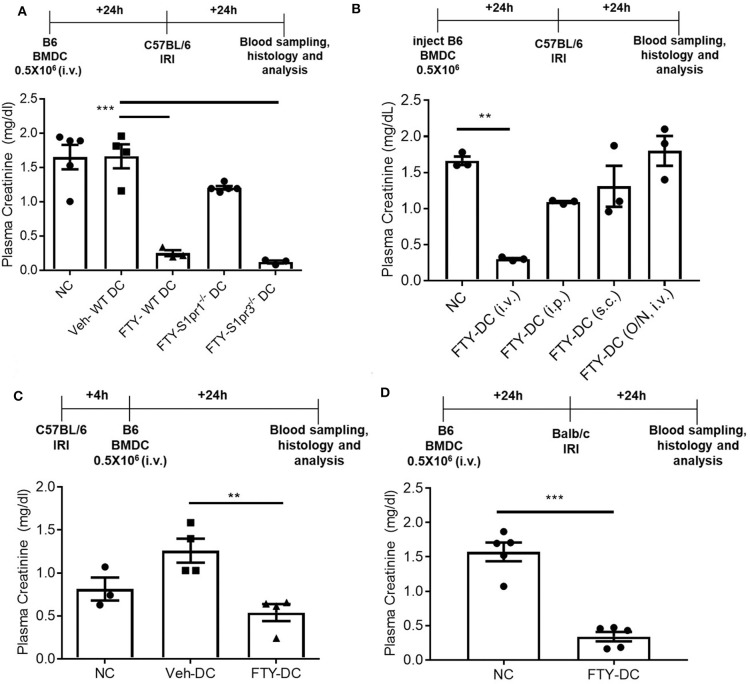
FTY-DC require S1pr1 on BMDC to protect kidneys from IRI. **(A)** Protocol for experimental setup. FTY720 is a ligand for four out of five S1P receptors. Plasma creatinine (PCr, mg/dL) was measured 24 h after IRI. We tested if S1pr1 or S1pr3 were requited for FTY720 dependent regulatory DC phenotype. BMDCs were propagated from either C57BL/6 WT, *CD11cCreS1pr1*^*fl*/*fl*^ (*S1pr1*^−/−^ DC), or *S1pr3*^−/−^ and treated with FTY720. FTY-*S1pr1*^−/−^ DC do not protect kidneys from IRI. As demonstrated in our earlier published studies and again confirmed, transfer of *S1pr3*^−/−^ DC with or without FTY720 significantly protect kidney from IRI. These studies suggest only *S1pr1* are necessary for FTY720 dependent regulatory DC phenotype. Next, we tested if the route of delivery was important for FTY-DC induced protection from kidney IRI. **(B)** Protocol for experimental setup. Plasma creatinine was measured 24 h after IRI. FTY-DC were injected either intravenous (i.v.), intraperitoneal (i.p.), subcutaneous (s.c.) or as i.v. using Veh-DC that were acutely treated with FTY720 (overnight along with LPS). These studies suggest that FTY-DC only protect kidneys from IRI if injected via i.v. and the FTY-DCs must be propagated in presence of FTY720 at start of the BMDC culture. **p ≤ 0.01. **(C)** Protocol for experimental setup. Plasma creatinine was measured 24 h after IRI. In all studies presented we injected FTY-DC 1 day before kidney IRI. We also tested if FTY-DC could be injected after injury. Mice were treated with either NC, Veh-DC or FTY-DC 4 h after bilateral IRI. Mice injected with Veh-DC had higher PCr compared to NC mice and FTY-DC treated mice were significantly protected. **(D)** Protocol for experimental setup. Plasma creatinine (mg/dL) was measured 24 h after IRI. C57BL/6 FTY-DC induce protection in BALB/c mice and protect kidneys from ischemic injury. Data represent means ± SEM, Unpaired *t*-test [D], ***p ≤ 0.001; **p ≤ 0.01 and ***p ≤ 0.001, one-way ANOVA followed by Tukey's post-test.

FTY720 dependent protection is mainly due to its binding to S1P1 at low doses and potentially followed by S1P3 at higher doses. It is unclear which receptor FTY720 may be signaling through to induce such protection from IRI. To determine the mechanisms mediating downstream effects of FTY720, *CD11cCreS1pr1*^*fl*/*fl*^ (*S1pr1*^−/−^-DC), and *S1pr3*^−/−^ mice were used to harvest BMDCs and propagate in presence of FTY720. C57BL/6J mice were injected with half million LPS activated FTY-*CD11cCre* (WT) DC, FTY-*S1pr1*^−/−^ DC or FTY-*S1pr3*^−/−^ DCs 1 day before bilateral kidney IRI. FTY-*CD11cCre* (WT) DC and as expected from our previous studies (12, 13) *S1pr3*^−/−^ DCs treated with FTY protected mice kidneys from injury. The protection was abrogated in mice treated with FTY-*S1pr1*^−/−^ DC (Figure 5A). Interestingly, the protection by FTY-DCs is lost if BMDCs are administered either through intraperitoneal or subcutaneous injections and if DCs are only treated acutely (overnight) with FTY720 (Figure 5B). Therapeutic use of FTY-DCs is maintained even if given 4 h after kidney ischemia (Figure 5C) or if tested in allogeneic transfer experiments (C57BL/6J DCs to BALB/c) in mice (Figure 5D). In allogeneic transfer studies the BMDCs were not activated with LPS prior to transfer and equally protect mice kidneys from IRI. This could be due to involvement of adaptive immunity with FTY-DC.

### Mitochondria Function Is Critical in FTY-DC Dependent Protection

**Figure 6:**
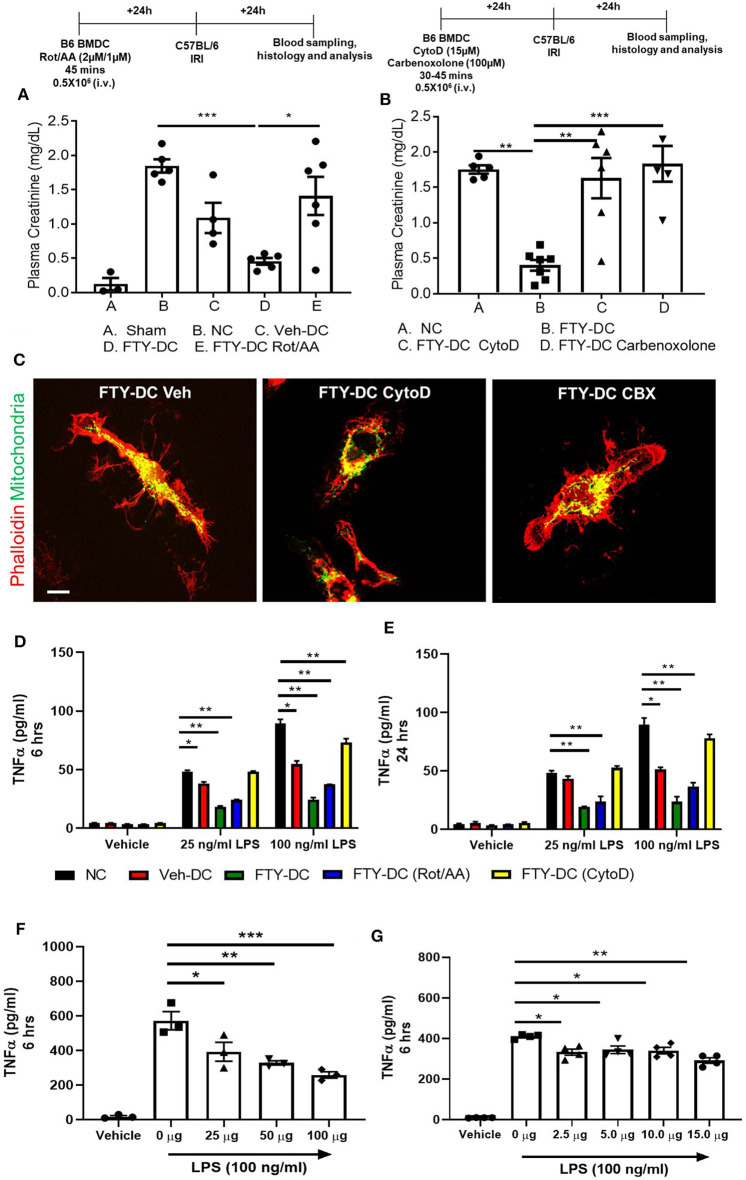
Inhibition of FTY-DC mitochondrial function abrogates protection from kidney IRI. Protocol for experimental setup. **(A)** Plasma Creatinine (PCr). Mice were i.v. injected with 0.5 × 10^6^ DCs (Veh-DC or FTY-DC) and as control no cells (NC) 1 day before bilateral kidney IRI. A group of mice were injected with FTY-DC that were treated with rotenone and antimycin A (Rot/AA). **(B)** Protocol for experimental setup. Plasma Creatinine (PCr). Mice were i.v. injected with 0.5X10^6^ DCs (FTY-DC) and as control no cells (NC) 1 day before bilateral kidney IRI. Two additional group of mice were injected with FTY-DC that were treated with cytochalasin D (Cyto D) or carbenoxolone (CBX). **(C)** Immunofluorescence of *CD11cCrePham*^*fl*/*fl*^ FTY-DC (green, mitochondria) and labeled with phalloidin (red, actin) that were treated with Cyto D or CBX. Scale bar, 20 μm. **(D,E)** Mice were i.v. injected with 0.5 × 10^6^ DCs (Veh-DC or FTY-DC) or (FTY-DC treated with Rot/AA or Cyto D) and as control no cells (NC) 1 day before splenocytes were harvested and treated *ex vivo* with LPS (25 or 100 ng/ml) for 6 or 24 h and supernatant was analyzed by Elisa for TNFα. **(F)** Mice were i.v. injected with various amounts of isolated mitochondria (0–100 μg/mouse). Spleen was harvested 1 day after mitochondria injections and single cells suspensions were treated *ex vivo* with 100 ng/ml LPS for 6 h and supernatant was analyzed by Elisa for TNFα. **(G)** Spleen from (0 μg mito) treated mouse was harvested and incubated with various amounts of isolated mitochondria (0–15 μg/well) for 1 day before stimulating with 100 ng/ml LPS for additional 6 h and supernatant was analyzed for TNFα. Data represent means ± SEM, *p ≤ 0.05, **p ≤ 0.01, and ***p ≤ 0.001, one-way ANOVA followed by Tukey's post-test.

Next, we tested if transferred FTY-DC (1) actively participate in transferring mitochondria to recipient splenic cells through actin polymerization, (2) required intact functional mitochondria, or 3) if active production of mitochondria (ATP) was involved in FTY-DC dependent protection from injury. LPS treated FTY-DC were treated with Rotenone (inhibitor of mitochondrial electron transport) and Antimycin A (inhibitor of cellular respiration) (Rot/AA; 2 μM/1 μM). As control LPS treated Veh-DC and FTY-DC were treated with equivalent volumes of DMSO. Treating FTY-DC with Rot/AA to reduce mitochondrial functions abrogated the protection from kidney IRI (Figure 6A). Similarly, treating FTY-DC with either Cytochalasin D (CytoD; 15 μM, an inhibitor of actin polymerization) or carbenoxolone (CBX; C4790; Sigma-Aldrich; 100 μM, non-specific inhibitor of gap junctions) abrogated the protection compared to FTY-DC treated group (Figure 6B). *CD11cCrePham*^*fl*/*fl*^ BMDCs were used to check if FTY-DC treated with either Rot/AA, CytoD, or CBX had differences in homing *in vivo* after injection. There were no statistically significant changes in number of cells found in the recipient spleen with either of the three inhibitors (data not shown). Additionally, no changes in cell viability (Annexin V and 7-AAD) were observed in FTY-DC treated with either Rot/AA, CytoD or CBX (data not shown). The structural changes in FTY-DC were also analyzed after CytoD and CBX treatment after 24 h. Compared to Veh treated FTY-DC (Figure 6C, CytoD treated cells have changes in actin (Figure 6C,) and no changes were observed in actin with CBX treatment (Figure 6C, right panel). To test if the FTY-DC-dependent transfer of mitochondria induced a change in cellular responses in spleen we harvested spleen from mice that were treated with either NC (1x PBS), Veh-DC, FTY-DC, FTY-DC (Rot/AA), or FTY-DC (CytoD) 24 h after injection. Total splenic single cell suspensions (~500,000/well) were treated with LPS (25 ng/ml or 100 ng/ml) for either 6 or 24 h from the 5 groups. Splenocytes from FTY-DC (green) treated mice had significantly lower levels of TNF-α at 6 h compared to NC (black) or Veh-DC (red) treated mice (Figures 6D,E). Splenocytes cultures from mice treated with FTY-DC (Rot/AA) (blue) or FTY-DC (CytoD) (yellow) had higher levels of TNF-α compared to FTY-DC treated mice. Possibly due to less mitochondria transfers from [(Rot/AA) or (CytoD)] treated FTY-DC to splenocytes. We noted that splenocytes that were isolated from mice treated with Veh-DCs had significantly less production of TNFα with 100 ng/ml LPS, suggesting that Veh-DCs also donate mitochondria to splenocytes (as also shown in Figure 3) although to lesser extent compared to FTY-DCs. To test the hypothesis that increasing mitochondria numbers as expected with FTY-DCs are responsible for inhibitory effect on TNFα production from LPS treated splenocytes, we treated mice with different doses of healthy isolated mitochondria. To test if dose dependent uptake of mitochondria was responsible for inducing an anti-inflammatory phenotype in splenocytes, we injected mice with various amounts of isolated labeled mitochondria and found that injected mitochondria signal was mainly found in splenic macrophages as early as 30 min after injection (data not shown). In order to check if injected mitochondria are found in recipient mouse spleen, we either used *CD11cCrePham*^*fl*/*fl*^ BMDCs or human HEK293 cells to isolate mitochondria in separate experiments. Mice injected with mitochondria isolated from *CD11cCrePham*^*fl*/*fl*^ BMDCs were imaged after 24 h after injection (i.v.). As shown in Figure 3, in these mice, systemically injected labeled mitochondria in spleen was predominantly found in F4/80^+^ and CD169^+^ macrophages (data not shown). However, unlike data shown in Figure 3, signal associated with systemically injected mitochondria was also found in various other tissues including kidneys (data not shown). Using 50 μg mitochondria isolated from human HEK293 cells we were able to check with RTPCR the expression using human and mouse mtDNA primers in various tissues over time. In spleen relative levels of h-mtDNA/m-mtDNA expression increased as early as 30 min after injected and was higher at 24 h after injection compared to uninfected mice (data not shown). Mice were intravenously injected with various amounts (0–100 μg/mouse) of isolated mitochondria isolated from mouse liver. Splenocytes from mitochondria treated mice were cultured 24 h after injection and stimulated with LPS for 6 h. Total splenic single cell suspensions (~100,000/well) were treated with 100 ng/ml LPS for 6 h. Mice treated with mitochondria have a significant dose dependent decrease in TNFα production compared to control (0 μg mitochondria) treated mice (Figure 6F). Additionally, treatment of control splenocytes (0 μg mitochondria) with isolated mitochondria *ex vivo* (0–15 μg/well) 1 day before treatment with LPS also significantly reduced TNFα in 6 h cultures in a dose dependent manner (Figure 6G).

### FTY-DC Are More Efficient Mitochondria Donors Compared to Veh-DC

**Figure 7:**
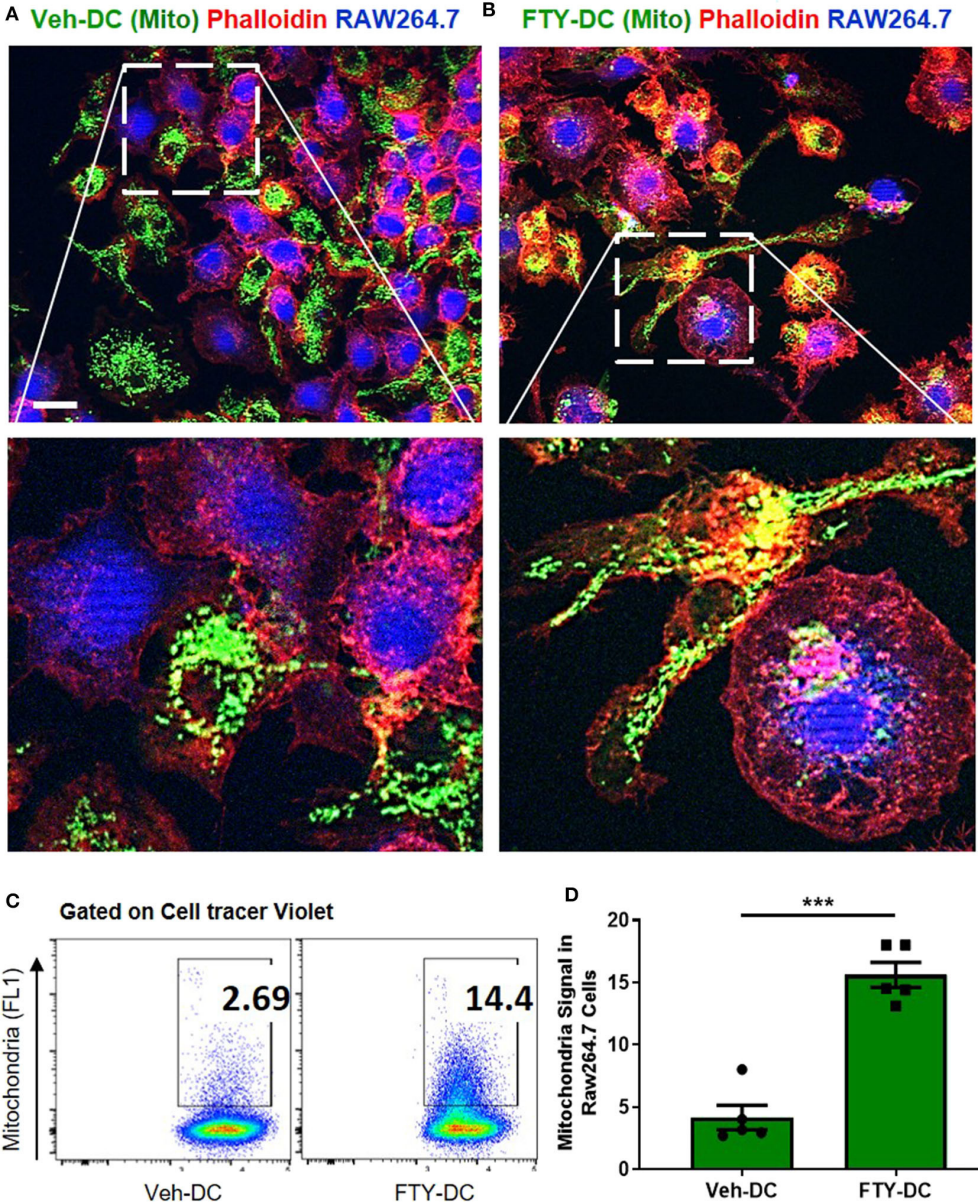
Dendritic cells transfer mitochondria to macrophages. **(A)** Co-culture of 1:1 of macrophages (RAW264.7, blue, cell tracer violet) and *CD11cCrePham*^*fl*/*fl*^ BMDCs (Veh-DC, green, mitochondria) and labeled with phalloidin (red, actin) was done after 24 h. **(B)** Co-culture of 1:1 of macrophages and *CD11cCrePham*^*fl*/*fl*^ BMDCs (FTY-DC). Scale bar, 20 μm. **(C,D)** Flow cytometry of co-culture and semi-quantitative analysis after 24 h was done by gating on cell tracer violet positive RAW264.7 cells. Data represent means ± SEM, ***p ≤ 0.001. One of three experiments is shown.

Using coculture of BMDCs and RAW264.7cells (mouse macrophage cell line), we tested the efficiency of FTY- vs. Veh-DCs to donate mitochondria. Prior to setting up the co-culture, RAW264.7 cells were labeled blue using CellTrace^TM^ Violet (CT-Violet) proliferation dye. All analysis (imaging and flow cytometry) was done after 24 h. Compared to Veh-DCs, coculture of RAW264.7 cells with FTY-DCs had more transfer of mitochondria by immunofluorescence (Figures 7A,B) and quantification by gating on CT Violet (RAW264.7) and evaluating the amount of donor (Pham) green mitochondria signal (Figure 7C). Co-culture of RAW264.7 (Blue, CT Violet) cells with FTY-DC (green mitochondria) have significantly more mitochondria donation compared to Veh-DC co-cultures (Figure 7D).

### Uptake of Healthy Mitochondria by Macrophages Induce a Less Immunogenic Phenotype

**Figure 8:**
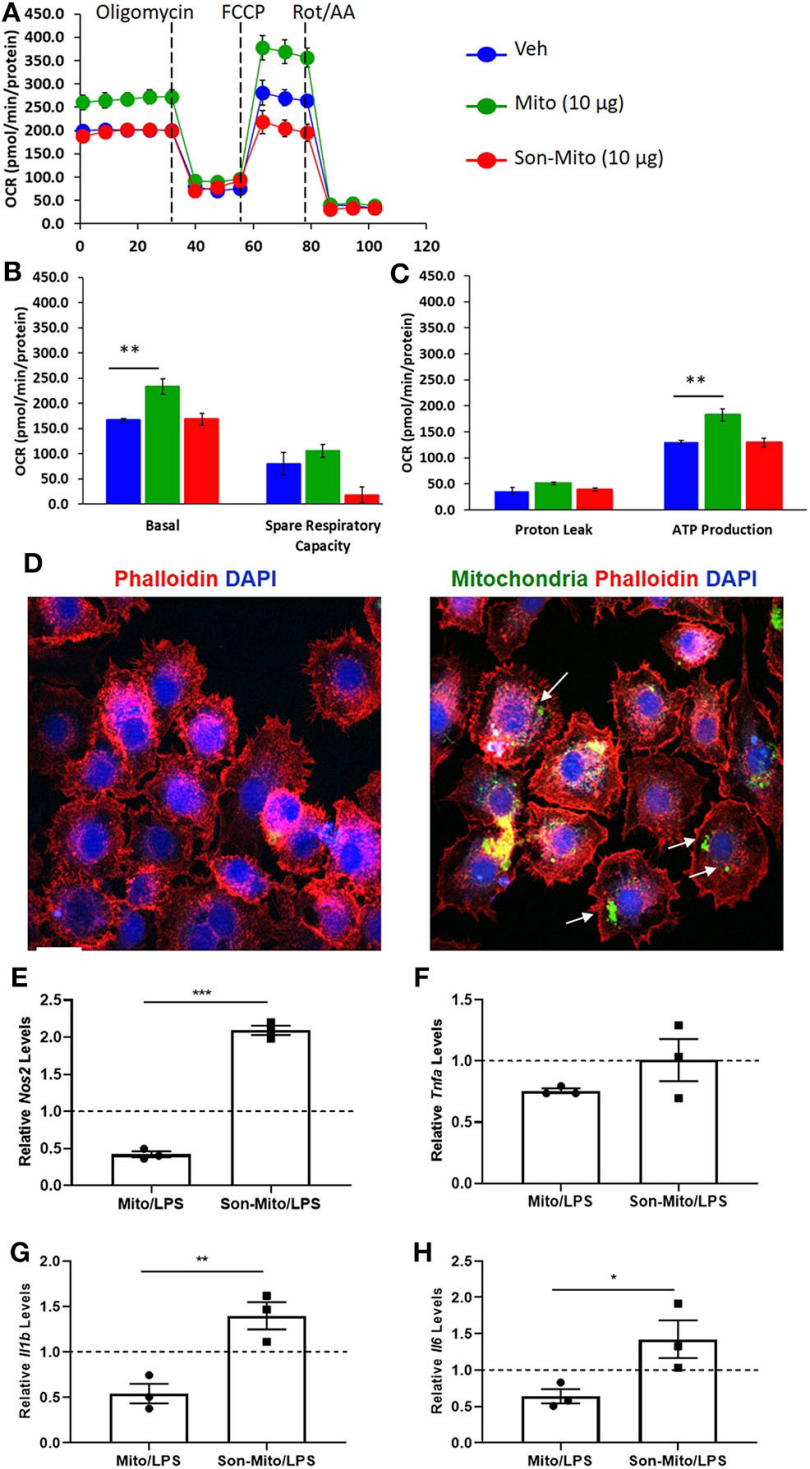
Mitochondria donation from DCs induce anti-inflammatory phenotype in macrophages. **(A)** Seahorse analysis of RAW264.7 cells treated with healthy mitochondria (Mito) or structurally unhealthy (Son-Mito) for 24 h. **(B,C)** Oxygen consumption rate of RAW264.7 cells measure basal, spare respiratory capacity, proton leak and ATP production. The assay was normalized to total protein. **(D)** RAW264.7 cells were treated with 0 or 10 μg/well mitochondria isolated from 8 day old *CD11cCrePham*^*fl*/*fl*^ BMDCs. White arrows point to labeled mitochondria that appears perinuclear in RAW264.7 cells. Scale bar, 20 μm. **(E–H)** 10 μg/well mitochondria (with and without sonication) was added to RAW264.7 cells for 24 h prior to treatment with 100 ng/ml LPS for additional 24 h. Gene expression of *Nos2, Tnfa, Il1b*, and *Il6* was analyzed. Data calculated as fold increase over Veh/LPS treated cells, shown as dash line. Data represent means ± SEM of triplicates, **p ≤ 0.01, one-way ANOVA followed by Tukey's post-test and Unpaired *t*-test **(E–H)**, *p ≤ 0.05, **p ≤ 0.01, and ***p ≤ 0.001. One of three experiments is shown.

To determine if uptake of healthy mitochondria by RAW264.7 cells changes their responses to LPS, we repeated the above study with isolated mitochondria rather than DC-dependent donation. BMDCs were again propagated from *CD11cCrePham*^*fl*/*fl*^ mice and mitochondria was isolated from 8 day old Veh-DCs. RAW264.7 cells were treated with 10 μg/well of isolated mitochondria for 24 h. Some of the treated RAW264.7 cells were used for seahorse analysis and rest were treated with 100 ng/ml LPS for additional 24 h for gene analysis. As control, equal amounts of sonicated (Son) mitochondria (Son-Mito) were added in separate wells for 24 h. Treatment of RAW264.7 cells with healthy mitochondria significantly induced an increase in basal oxygen consumption rate (OCR) and ATP production compared to vehicle treated cells and use of Son-Mito abrogated these effects (Figures 8A–C). Mitochondria treated RAW264.7 cells were also analyzed for uptake of labeled mitochondria 24 h after incubation, the added mitochondria signal appears perinuclear in location (white arrows, Figure 8D). We next tested if addition of mitochondria regulated gene expression in RAW264.7 cells stimulated with LPS. Compared to untreated RAW264.7 cells (Veh/LPS), cells treated with healthy mitochondria had lower mRNA expression levels for *Nos2, Tnfa, Il1b*, and *Il16* after LPS stimulation (Mito/LPS). However, treatment of RAW264.7 cells with Son-Mito abrogated these inhibitory changes compared to healthy functional mitochondria (Figures 8E–H) treated cells. Data was calculated as relative fold changes compared to Veh/LPS treated cells (dash line, Figures 8E–H).

## Discussion

In the current study we demonstrated that immunosuppression and protection from kidney IRI induced by adoptive transfer of FTY-DC is dependent on the recipient spleen, DC-S1P1 and functional viability of transferred DC mitochondria. Furthermore, the protective effects of FTY-DC involve donation of mitochondria to splenic macrophages making them less immunogenic. In addition, our study for the first time to our knowledge demonstrates that BMDCs like mesenchymal stem cells (63) have the potential to donate mitochondria to induce an immunosuppressive phenotype in recipient cells.

### Dendritic Cells in Acute Kidney Injury

AKI is a major health burden without major pharmacological advances in its prevention or treatment (64). Additionally, current therapies for allograft rejection, cancer, or autoimmune diseases use non-specific immunosuppressive drugs that are associated with adverse side effects and are limited due to lack of antigen specific tolerance. DCs are a heterogeneous group of cells important in immunity or tolerance, and the idea of using tolerized DCs in cell-based therapy of cancer, autoimmune disease, and transplantation has been under investigation for the past 2 decades (65). However, most studies have focused on the induction of T cell–tolerogenic responses. Immune regulation of innate immune response via tolerogenic DCs is critically important in bridging innate and adaptive immunity and provides the foundation for use in transplant tolerance of allograft injury (66). Cell-based therapy using regulatory immune cells [Tregs (67), myeloid cells (68), or DCs (69, 70)] is a strategy that induces potential antigen-specific tolerance. Pharmacological or biological strategies induce regulatory or tolerogenic DCs (Tol-DC) (71), which are immature, maturation-resistant or alternatively activated cells that express low levels of MHC and co-stimulatory molecules. Compared with mature DCs, immature DCs interact actively with T cells and direct them into a regulatory response. Depletion of DCs significantly protects mouse kidneys from IRI (6, 13) and a dose-dependent increase in BMDC numbers exacerbates kidney injury (13), suggesting that DCs play a major role in inducing AKI. As our current and previously published studies demonstrate injected BMDCs accumulate in the spleen (Figure 3) after systemic infusion (72) and can persist for two weeks post-injection (73). In kidney IRI, DCs tolerized with an A_2A_R agonist (74) or DCs deficient of *S1pr3* (13) attenuated AKI. Our current study further demonstrates using *CD11cCrePham*^*fl*/*fl*^ mice to harvest DCs that transferred DCs can donate their mitochondria to recipient cells thus making them less immunogenic.

### Role of S1P Receptor Agonist (FTY720) in Kidney Injury and Dendritic Cells

S1P1 activation is important for maintaining cell viability; global deletion is embryonically lethal (75). We have previously demonstrated that the protective effect of S1P1 agonists FTY720 or SEW2871 in IRI (54) and cisplatin-induced nephrotoxicity (14, 49) was mediated by activation of S1P1 expressed on PT cells, independent of lymphopenia (14). Others have also shown that FTY720 can act as innate immune system immunomodulator that involves a role beyond its prominent effects on lymphocyte recirculation (76). In another study, using a mixed lymphocyte reaction (MLR), FTY720-treated human DCs exhibited reduced antigen presentation and altered cytokine production (77) and systemic injection with FTY720 was also found to block DC trafficking (78). Our current data (Supplemental Figure 1) and others have previously demonstrated that FTY720 alone does not affect the surface of BMDCs surface markers CD11c, MHCII, CD40, CD86, and indicates there is no change in viability. However, BMDCs propagated in presence of FTY720 do have immunosuppressive phenotype upon stimulation (LPS, CD40L or mixed lymphocyte reactions) and transfer of these immunosuppressive BMDCs confirms protection in various models CD80 (56, 77, 79, 80). In many of these studies the protective effects of FTY720 treated BMDCs was due to infusion of these immunosuppressive cells to block T cell responses. FTY-DCs in our study are immature compared to Veh-DCs after LPS stimulation and transfer of these FTY-DCs could potentially have regulatory responses on adaptive immunity. In our experiments we did observed a higher number of Tregs in spleen of FTY-DC treated mice but the exact mechanism of this is yet to be determined.

### Role of Mitochondria in Dendritic Cells and Macrophages

DC and macrophage functions are regulated by mitochondrial metabolism. Type 1 macrophages (81, 82) and immunogenic DCs have high glycolytic rates (62). The activation of DCs or macrophages by several TLR agonist (LPS or CpG) leads to rapid increase in glycolysis followed by decrease in OXPHOS and mitochondrial membrane potential (62, 83, 84). Some role for mitochondria has been demonstrated with DCs treated *in vitro* with vitamin D, these DCs have increased OXPHOS, mitochondrial mass and mROS production (85), similar to what we observe with FTY-DCs. Although the mechanistic details of how glycolysis and mitochondria metabolism controls DC function are unknown our data suggests FTY-DC continue to rely of mitochondria OXPHOS and this could contribute to their less immunogenic phenotype. Multiple mechanisms may exist by how FTY-DC protect kidneys from IRI, one possible mechanism is through mitochondria donation. Our current data indicates that transferred FTY-DCs are more efficient at transferring mitochondria to recipient splenocytes, mainly macrophages (CD169^+^ or F4/80^+^). The exact mechanism of how mitochondria get transferred to macrophages is unknown, however use of either actin polymerization inhibitor Cyto D or non-specific gap junction inhibitor CBX abrogated FTY-DC dependent protection. Treatment with Cyto D or CBX did not change the viability or trafficking of FTY-DC *in vivo* (data not shown). *in vivo* transferred Veh-DC also donate mitochondria to splenocytes however to a lesser extent compared to FTY-DC. This was clear as stimulation of splenocytes isolated from mice 24 h after various DC infusion [Veh-DC, FTY-DC, FTY-DC (Rot/AA), or FTY-DC (CytoD)] had less TNF-α levels after *ex vivo* stimulation with LPS compared to NC splenocytes. TNFα was significantly lower in Veh-DC treated splenocytes compared to NC splenocytes at 6 h after LPS stimulation. Splenocytes that were from FTY-DC treated mice displayed most suppression in TNFα production, an effect that was partially lost if FTY-DC were pretreated with Rot/AA or Cyto D demonstrating a dependence upon mitochondrial function. These data indicate that uptake of naked/free mitochondria or DC-derived mitochondria in a dose dependent manner induces an anti-inflammatory phenotype, although the exact mechanism is currently unknown.

Our current findings indicate that DCs have the potential to donate mitochondria to induce immunological changes in the recipient cells a protective mechanism previously shown to be employed by mesenchymal stem (86) and bone-marrow-derived stromal cells (87). As demonstrated in our earlier studies (12), the injected bone-marrow-derived DCs are predominantly found in the recipient spleen as early as 30 min and signal persist up to 72 h. More importantly using transgenic mice (that contain labeled Pham mitochondria) to propagate DCs (*CD11cCrePham*^*fl*/*fl*^) our study is the first to demonstrate in addition to homing to the spleen, injected DCs donate mitochondria to splenic macrophages. Compared to naïve DC, DCs propagated in presence of FTY720 (FTY-DC) are more efficient at donating mitochondria to recipient splenocytes mainly to macrophages. The exact mechanism of how FTY-DC donate mitochondria is currently unknown but does involve gap junctions and actin polymerization as treatment with inhibitors (Cyto D or CBX) abrogates the protection by FTY-DCs. Our current study analyzed the involvement of macrophage dependent innate adaptive immunity in FTY-DCs dependent protection. However, we did note that in spleen of FTY-DC treated mice there was an increase in labeling of white pulp CD4^+^FoxP3^+^ cells that was in addition to disrupted CD169^+^ labeling of the MZ, similar to as previously demonstrated using *S1pr3*^−/−^ DCs (12). Thus, in addition to donating mitochondria to splenic macrophages FTY-DCs could also regulate adaptive immune responses resulting in higher Treg cells.

Limitation of our current study using mouse kidney IRI model is that this model is acute (<2 days), thus it is possible that if mice are followed for longer time periods after infusion of FTY-DCs especially in allogenic transfers (C57BL/6J BMDCs→ BALB/c mice or inverse), we might have a significant change in adaptive immune responses including higher Treg numbers. Since FTY-DCs are immunogenically immature (low CD80, CD86, MHCII, and higher IL10) after LPS stimulation and injection of these FTY-DCs increases splenic Tregs, we are in process of testing if FTY-DCs can be used to delay rejection using allogenic mouse model of heterotopic heart transplant. Lastly, if higher mitochondria numbers in FTY-DCs indeed induce the protection we observed, it would be interesting to test if artificially increasing DC mitochondria numbers (mitochondria transplant) also have similar therapeutic advantage. This is especially important since as current study demonstrates, we must propagate DCs in presence of FTY720 from start of BMDC cultures, as acute (overnight) treatment of DCs with FTY720 does not protect kidneys from IRI.

In summary we have demonstrated that BMDCs can regulate innate immune response by donating mitochondria. The anti-inflammatory responses induced by FTY-DC are dependent on the spleen and presence of S1P1 receptors. In the spleen, FTY-DC donate mitochondria more efficiently compared to Veh-DC to splenic macrophages (F4/80^+^ and CD169^+^). Dose dependent uptake of mitochondria by splenic and RAW264.7 macrophages induces metabolic reprogramming that is a key driver of anti-inflammatory phenotype. We conclude that regulatory FTY-DC may be useful in kidney IRI as well as in other inflammatory states such as transplantation and autoimmune disorders.

## Data Availability Statement

The datasets generated for this study are available on request to the corresponding author.

## Ethics Statement

The animal study was reviewed and approved by University of Tennessee Health Science Center and University of Virginia Institutional Animal Care and Use Committees.

## Author's Note

The current abstract was previously presented as oral presentation at American Transplant Congress in 2019 and published online (https://doi.org/10.1111/ajt.15405).

## Author Contributions

AB conceived the idea, designed the experiments, analyzed data, and wrote the manuscript. AB, TR, LH, and KS supervised the kidney ischemia experiments and critically reviewed the data and manuscript. AB, TR, LH, KS, CeK, and CaK performed experiments and prepared the figures. MN helped with western blots. AB, VM, DM, JE, and LM reviewed and edited the manuscript. All authors contributed to the article and approved the submitted version.

## Conflict of Interest

The authors declare that the research was conducted in the absence of any commercial or financial relationships that could be construed as a potential conflict of interest.
