# Supplementary figures and images for: FTY720 Regulates Mitochondria Biogenesis in Dendritic Cells to Prevent Kidney Ischemic Reperfusion Injury
Source: Front Immunol. 2020 Jun 23;11:1278. doi: 10.3389/fimmu.2020.01278 (PMC7328774; doi:10.3389/fimmu.2020.01278)

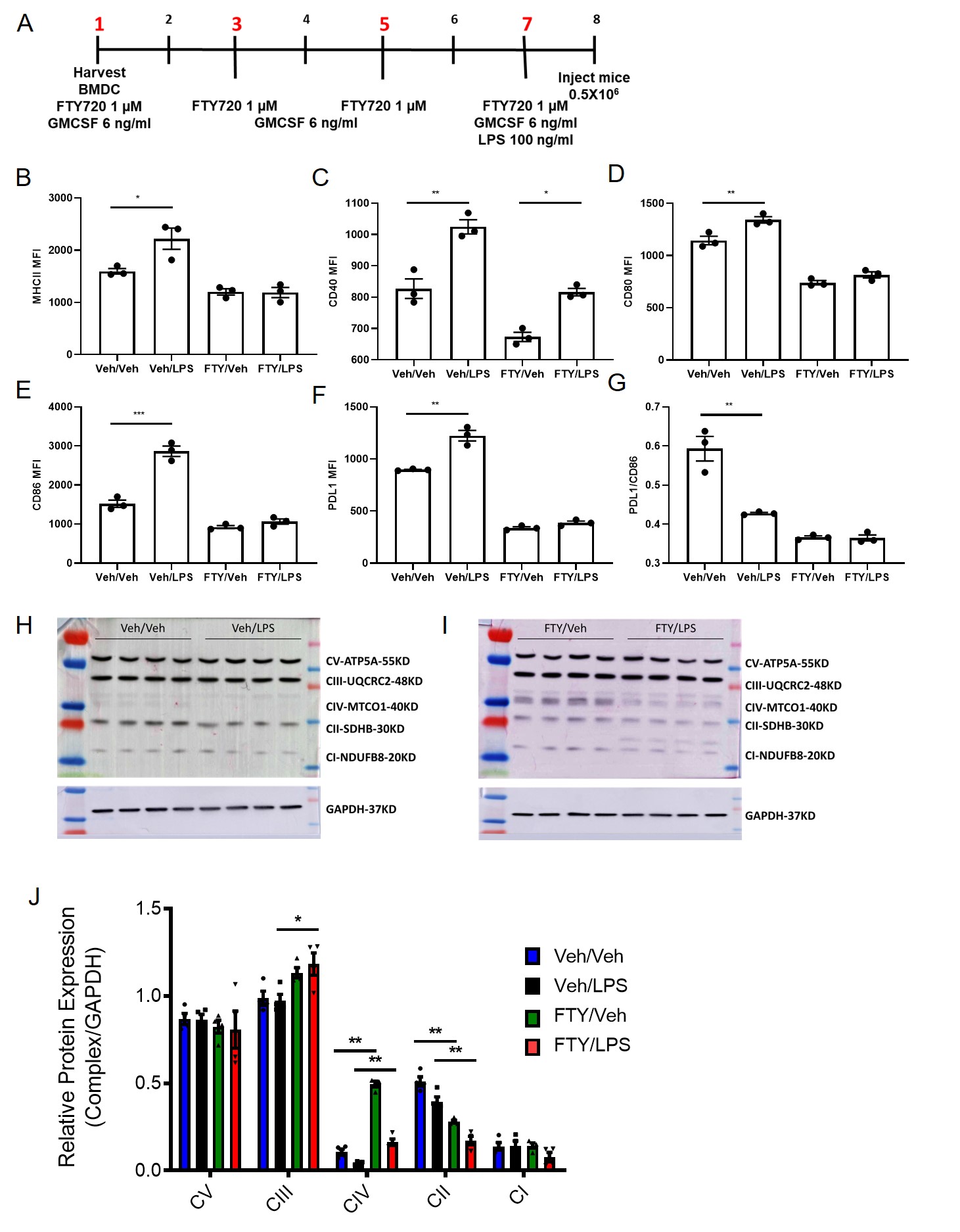

Supplement: Supplementary file 1 [file Image_1.JPEG]
