# Supplementary material for: FTY720 Regulates Mitochondria Biogenesis in Dendritic Cells to Prevent Kidney Ischemic Reperfusion Injury
Source: Front Immunol. 2020 Jun 23;11:1278. doi: 10.3389/fimmu.2020.01278 (PMC7328774; doi:10.3389/fimmu.2020.01278)
Supplement: Supplementary file 2 [file Presentation_1.pdf]

SUPPLEMENTAL FIGURE 1 FTY-DC are immature after LPS stimulation compared to Veh-DC. **(A)** Timeline of BMDC propagation with GMCSF (3 total treatments) and FTY720 (4 total treatments). 7-day old BMDCs either Vehicle or FTY720 treated were stimulated overnight with 100 ng/ml LPS. Flow cytometry of BMDCs, gated on live, singlets, $CD11c^+$ cells, graphs shown of MFI. **(B)** Expression of MHCII. **(C)** Expression of CD40. **(D)** Expression of CD80. **(E)** Expression of CD86. **(F)** Expression of PDL1 and **(G)** Ratio of PDL1/CD86. **(H)** Western blot analysis of Veh-DC stimulated overnight with and without 100 ng/ml LPS. **(I)** Western blot analysis of FTY-DC stimulated overnight with and without 100 ng/ml LPS. **(J)** Quantification of individual mitochondria complexes relative to GAPDH. Data represent means ± SEM, *$p \leq 0.05$, **$p \leq 0.01$, and ***$p \leq 0.001$ one-way ANOVA followed by Tukey's post-test.
